# The Role of Shyness and Fear of Negative Evaluation in Predicting the Mental Health of Academic Staff: A Cross-Sectional Study

**DOI:** 10.7759/cureus.73565

**Published:** 2024-11-12

**Authors:** Manijeh Firouzi, Saghar Azhdari, Shima Heybati, Farshid Fathy Karkaragh, Atiyeh Janbozorgi, Kosar Haghani

**Affiliations:** 1 Department of Psychology, University of Tehran, Tehran, IRN; 2 Department of Education and Psychology, Shahid Beheshti University, Tehran, IRN; 3 Department of Educational Management, Islamic Azad University West Tehran Branch, Tehran, IRN; 4 Department of Health Psychology, University of Tehran, Tehran, IRN; 5 Department of Psychology, Islamic Azad University North Tehran Branch, Tehran, IRN; 6 Department of Social Sciences, Texas Woman's University, Denton, USA

**Keywords:** academic staff, descriptive study, fear of negative evaluation, mental health, shyness

## Abstract

This study aimed to predict mental health based on shyness and fear of negative evaluation in academic staff. The research employed an applied methodology, utilizing quantitative and descriptive (correlational) methods for data collection. The study's temporal framework is cross-sectional. The statistical population comprises all educational staff at the Islamic Azad University, Ardabil branch, totaling 400 individuals employed in 2019. According to Morgan's table, the statistical sample includes 196 educational-administrative staff members, selected through stratified random sampling. For this research, we utilized three mental health questionnaires (depression, anxiety, and stress) by Lovibond and Lovibond, the Fear of Negative Evaluation Scale by Watson and Friend, and the Shyness Scale by Cheek and Buss to explore the relationships between variables. Pearson's correlation test and multiple linear regression analysis were employed. The results indicated that the relationships between mental health and its components (depression, anxiety, and stress) are positive and significant, with shyness and anxiety accounting for 54% of the variance in mental health. Additionally, the study found that the effects of shyness and fear of negative evaluation on depression, anxiety, and stress were 41%, 43%, and 39%, respectively. Based on these findings, we conclude that shyness and fear of negative evaluation can predict mental health in academic staff.

## Introduction

How people adapt in various fields can affect their lives. This includes academic, social, and work areas. These aspects are independence, acquiring new skills, communicating with the opposite sex, and decision-making [1]. Poor and inappropriate performance, given individual differences, can raise stress and anxiety [2]. Consequently, this process in people's lives can turn into a period of increased risk for developing mental health problems [3]. Recent research shows that 50% of university staff, professors, and students may have psychological disorders [4].

Mental health is a multifaceted concept encompassing various aspects of health. The World Health Organization [5] defines it as “a state of health in which an individual realizes his or her abilities, can cope with the normal stresses of life, can work productively and fruitfully, and can contribute to his or her community.” Another view, from Foster et al. [6], defines mental health as a state of psychological health that fosters personal growth and development. It empowers individuals to adapt to their surroundings and thrive in their environment. Those with good mental health can manage and overcome negative emotions. Also, their systems work optimally. This prevents stress-related physical symptoms [7]. But sometimes, for people who cannot control their stress, this leads to drug use [8]. Additionally, childhood traumas impact stress and anxiety in academic settings [9].

Fear of negative evaluation (FNE) is a key factor affecting mental health [10]. It is a strong, constant fear of social situations. This is especially true for those with unfamiliar people who might judge them [11]. This fear comes from worries about negative judgments. It leads to withdrawal from social interactions. This withdrawal can cause isolation, depression, and shyness [12]. In relationships, the fear of negative judgment creates an internal barrier. It affects social-psychological phenomena, such as conformity and social anxiety [13]. These consequences ultimately contribute to poorer mental health.

Shyness is a major mental health issue. It is excessive self-consciousness and anxiety in social situations [14]. Shy people often have social anxiety. It makes them reluctant to interact with strangers. Shy people are overly cautious in social situations. This fear of self-expression makes them sensitive to others' reactions. This sensitivity can show as signs of anxiety, like an increased heart rate, facial flushing, and other symptoms [15].

Although previous research has shown the importance of mental health for university staff at the Islamic Azad University of Ardabil, there is still a significant research gap regarding the relationship between shyness, FNE, and mental health outcomes in this population. Given the importance of mental health to university employees, few studies have been done on this topic. It is notable because their mental health can be affected by factors like FNE, shyness, and body esteem. For example, Naz found that social negativity and body acceptance predict life satisfaction [16]. They have a positive and significant effect. In another study, Vasudeva [17] found a significant and positive correlation between shyness and depression symptoms. Swami [18] showed that body image is related to dating anxiety. Furthermore, Liu et al. [19] discovered a correlation between elevated social anxiety and the worry of receiving a poor assessment. Despite these realizations, little research has been done on the exact relationships between shyness, FNE, and mental health among university employees. By examining the relationship between shyness and the fear of receiving an inadequate rating and mental health in university staff members, this study aims to fill this gap. This study seeks to provide significant understanding that can shape solutions and academic support systems by investigating these relationships.

## Materials and methods

This research is “applied” in terms of purpose and “descriptive correlation” in terms of information-gathering methods. Data were collected through a self-report questionnaire at a point in time. The statistical population of this research includes all the educational-administrative staff of the Islamic Azad University, Ardabil branch (400 people) who were employed in 2019, of which 400 people were selected as participants based on Morgan's table (for more information on participant selection, see Appendix 4). The university is considered a large educational environment in which there are employees with unique characteristics that cause their heterogeneity, but if it is possible to divide this large educational environment into several classes and departments such as the education department, research department, selection department, The development of human resources and faculty members was divided in order to increase the level of homogeneity of employees, it is possible to conduct research in these environments and make practical and research uses of their results. Therefore, in this research, the participants were selected using a stratified probability sampling method.

The following are the requirements for inclusion in this study: participants must be employees of Ardabil Azad University, have good mental health, and answer all questionnaire questions accurately and clearly. A mental or psychiatric illness, giving evasive or partial answers to the questionnaire, and not being connected to Azad University's Ardabil branch are among the exclusion grounds. The goals of the study, the participants' freedom to withdraw at any time, and the precautions taken to protect the privacy of their answers were all explained in detail to the participants. Before data collection, informed consent was obtained from each participant, confirming their voluntary participation and comprehension of the research procedure.

Three validated questionnaires were used to assess the study variables - the Depression, Anxiety, and Stress Scale (DASS-21) by Lovibond and Lovibond (1995), The Short Form of the Fear of Negative Evaluation Scale by Watson and Friend (1969), and the Cheek and Buss Shyness Scale (1983) [18-20]. Due to the number of variables under investigation, data collection was facilitated through the administration of these three questionnaires, each of which is explained below.

Psychological Health Scale (depression, anxiety, and stress)

This questionnaire, developed by Lovibond and Lovibond (1995), comprises 21 items divided into three subscales (depression, anxiety, and stress), with seven questions in each subscale [18]. Each of the DASS subscales includes seven questions, and the final score for each subscale is obtained by summing the scores of the related questions (Table 1). Each question is scored from 0 (does not apply to me at all) to 3 (extremely applies to me). Since the DASS-21 is a shortened form of the original scale (42 questions), the final score for each subscale should be doubled. By referring to Table 1, the severity of the symptoms can be determined (Lovibond and Lovibond, 1995). The validity and reliability of this questionnaire have been established in various contexts. Antony et al. reported high internal consistency (Cronbach's alpha) for the subscales [21]: 0.97 for depression, 0.92 for anxiety, and 0.95 for stress. In Iran, the validity and reliability of this questionnaire were examined by Samani and Jokar, who reported test-retest reliability for the depression, anxiety, and stress scales as 0.80, 0.76, and 0.77, respectively, along with Cronbach's alpha values of 0.81, 0.74, and 0.78, respectively [22-23].

**Table 1:** Severity of each subscale of depression, anxiety, stress scale (DASS) and their related questions

Subscales Indicators		Stress	Anxiety	Depression
Questionnaire questions		18،14،12،11،8،6،1	20،19،15،9،7،4،2	1،17،16،13،10،5،3
Intensity	Normal	0-14	7-0	9-0
	Mild	15-18	8-9	10-13
	Rage	19-25	10-14	14-20
	Intense	26-33	15-19	21-27
	Very intense	33	20	28

The Short Form of the Fear of Negative Evaluation Scale

This is a self-report scale designed by Watson and Friend (1969) and includes 12 items. The way to score the options in the FNE questionnaire is as follows: from the 5-point Likert scale (I am not like this at all = 1, I am a little like this, I am almost like this = 3, I am like this = 4, I am strongly like this = 5) was used. Statements 2, 4, 7, and 10 are scored in reverse, the total score is the sum of the scores of 12 statements, which is in the range of 12 to 60. A higher score indicates a high FNE in the individual and a low score and close to 12 indicates a low fear of the negative evaluation of others in the individual.

In 1969, Watson and Friend tested the reliability coefficients of this scale by retest method and Cronbach's alpha, respectively. 0.78 and 0.94 and its concurrent validity has been reported as 0.6 by calculating its correlation coefficient with the general anxiety scale [19]. The scale of FNE in Iran was translated and prepared for implementation by Mehrabizadeh Artman, Najarian, and Baharlu in 2018. These researchers have reported the reliability coefficient of this scale with Cronbach's alpha method of 0.86. [24].

Cheek and Buss Shyness Scale

This scale comprises 20 items developed by Cheek and Buss (1983) to assess shyness. Respondents rate their level of shyness for each item on a 5-point Likert scale (ranging from 1 for “completely disagree” to 5 for “completely agree”). Items 4, 7, 10, 13, 16, and 19 are reverse-scored. In this questionnaire, the highest score a person can get is 70 and the lowest score is 14. Obtaining a score close to 70 indicates a low level of shyness and obtaining a score close to 14 indicates a high level of shyness and shyness. The score of each of the subscales is calculated by summing the points of the questions of each of the subscales. The scale's psychometric properties have been evaluated in the Iranian population. Mozaddeh et al. reported a reliability coefficient of 0.89 using Cronbach's alpha, indicating good internal consistency [25]. Additionally, factor analysis supported the scale's construct validity, meaning it measures what it is intended to measure (shyness) in this population.

Information collection process

The research unit of Islamic Azad University, Ardabil branch granted the necessary permissions to publish the questionnaires to gather data for this cross-sectional study. Then, the questionnaires were sent as an online link in the Telegram channel belonging to the employees of Islamic Azad University, Ardabil branch. The employees' discretion was used. The questionnaires were designed so that the connection would automatically be blocked once the number of employees who could complete them reached the quorum, which was 196. Following the completion of the data-collecting phase, the data was examined by the guidelines provided for scoring each questionnaire.

The collected data were examined using a combination of descriptive and inferential statistical techniques. Descriptive statistics, including means, standard deviations, and frequency distribution tables, were produced using SPSS version 16 (IBM Corp., Armonk, NY). These statistics provide a summary of the data and highlight trends in the way that significant variables are distributed. For inferential statistics, Pearson correlation and multiple linear regression analysis were employed. These methods are employed to evaluate the connections among the research variables and, in the end, forecast how shyness and the dread of receiving a poor grade affect university staff members' mental health.

## Results

According to the statistical analysis related to Table 2 in this research, 65.30% (128 people) of the samples are men and 34.69% (68 people) are women. Most of these people have a bachelor's degree, 40.3% (79 people) have this level of education, while the least of them, 5.6% (11 people) have a bachelor's degree. Also, 87.8% (172 people) of the sample are administrative staff and 12.2% (24 people) are faculty members. The average work experience of the participants is 8.23 ​​years, and the highest amount of work experience is one year. The highest percentage of participants, 57.65% (113 people), are in the category of one to six years of work experience, while the lowest percentage, 4.08% (eight people), have 25 to 30 years of work experience. According to the results, the data related to employment history does not follow the normal distribution. In terms of marital status, 53.1% (104 people) of the sample are single and 46.9% (92 people) are married. In terms of health, 97.4% (191 people) of the sample are healthy and 2.6% (five people) have illness or disability. Finally, the average age of the participants is 34.52 years, the youngest is 25 years old and the oldest is 59 years old.

**Table 2:** Characteristics of participants

Variables	Subcomponents and indicators	Values	Percent	Frequency
Gender	Man	-	65.30	128
	Woman	-	34.69	68
Degree	Under diploma	-	5.6	11
	Diploma	-	7.7	15
	Associate degree	-	4.1	8
	Bachelor	-	40.3	79
	Master's degree	-	33.2	65
	Ph.D.	-	9.2	18
Job position	Administrative staff	-	87.8	172
	Faculty member professors	-	12.2	24
Marital status	Single	-	53.1	104
	Married	-	46.9	92
Health status	Healthy	-	97.4	191
	Having an illness or disability	-	2.6	5
Age	Minimum	20.00	-	-
	Maximum	59.00	-	-
	Mean	34.5153	-	-
	Std. Deviation	8.90144	-	-

The statistical analysis conducted for this study indicates that 34.69% (68 persons) and 65.30% (128 people) of the sample are women. The majority of these people - 40.3%, or 79 - have a bachelor's degree, while the smallest percentage - 5.6%, or 11 people - have only completed their undergraduate studies. Furthermore, faculty members makeup 12.2% (24 persons) and administrative personnel make up 87.8% (172 people) of the sample. The participants had an average of 8.23 years of work experience, with one year being the most prevalent experience level. Of all the participants, 57.65% (113) have between one and six years of job experience, while the smallest group, 4.08% (eight people), has between 25 and 30 years of experience. The results demonstrate that there is no normal distribution for the employment history data. Of the sample, 104 people, or 53.1%, are single, and 92 people, or 46.9%, are married. In terms of health, 2.6% (five persons) of the sample have illnesses or disabilities, compared to 97.4% (191 people) who are in good health. Lastly, the participants' average age is 34.52 years; the youngest is 25 years old, and the oldest is 59 years old.

**Figure 1:**
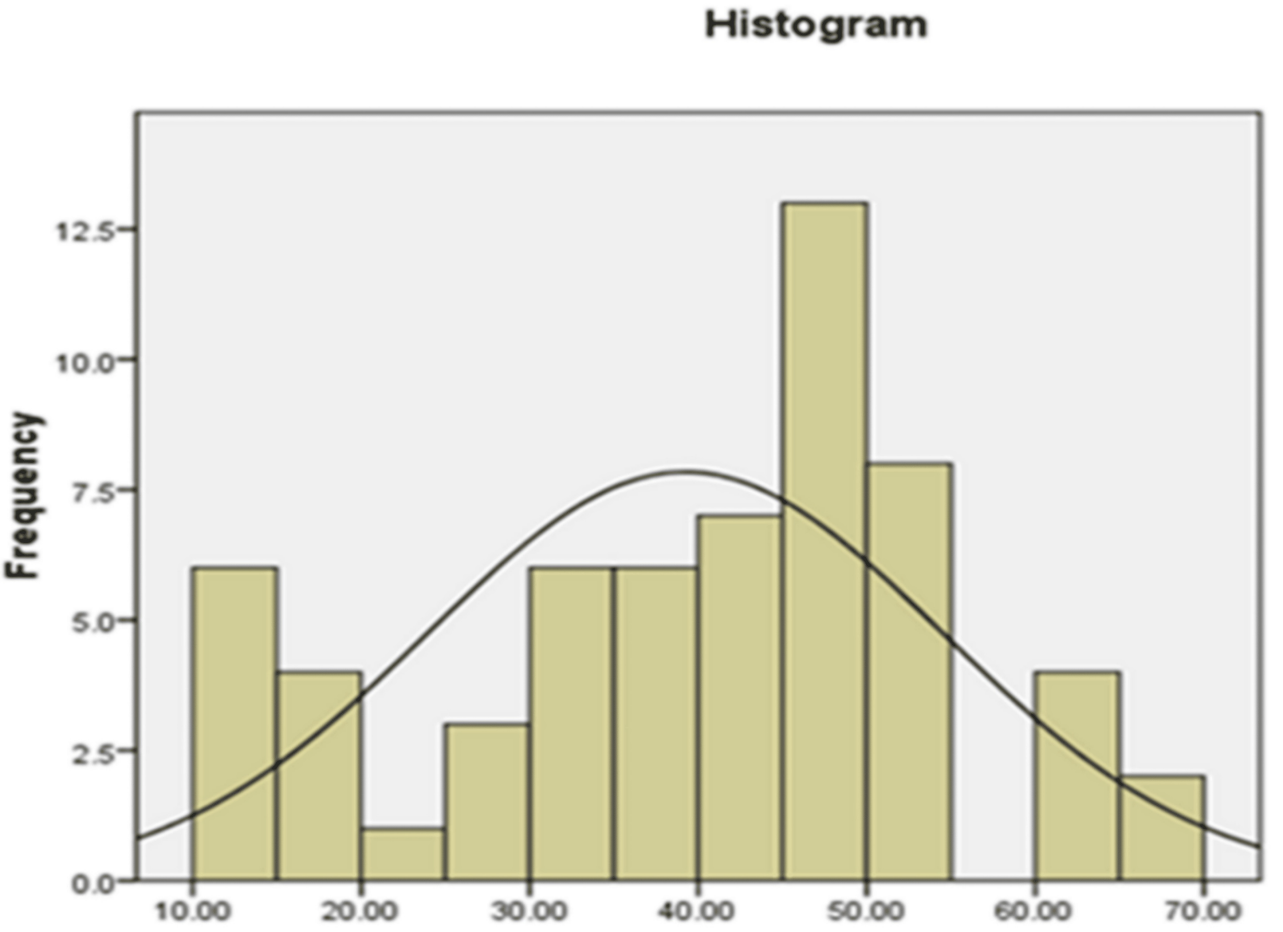
Histogram software to check the state of data distribution in mental health changes

**Figure 2:**
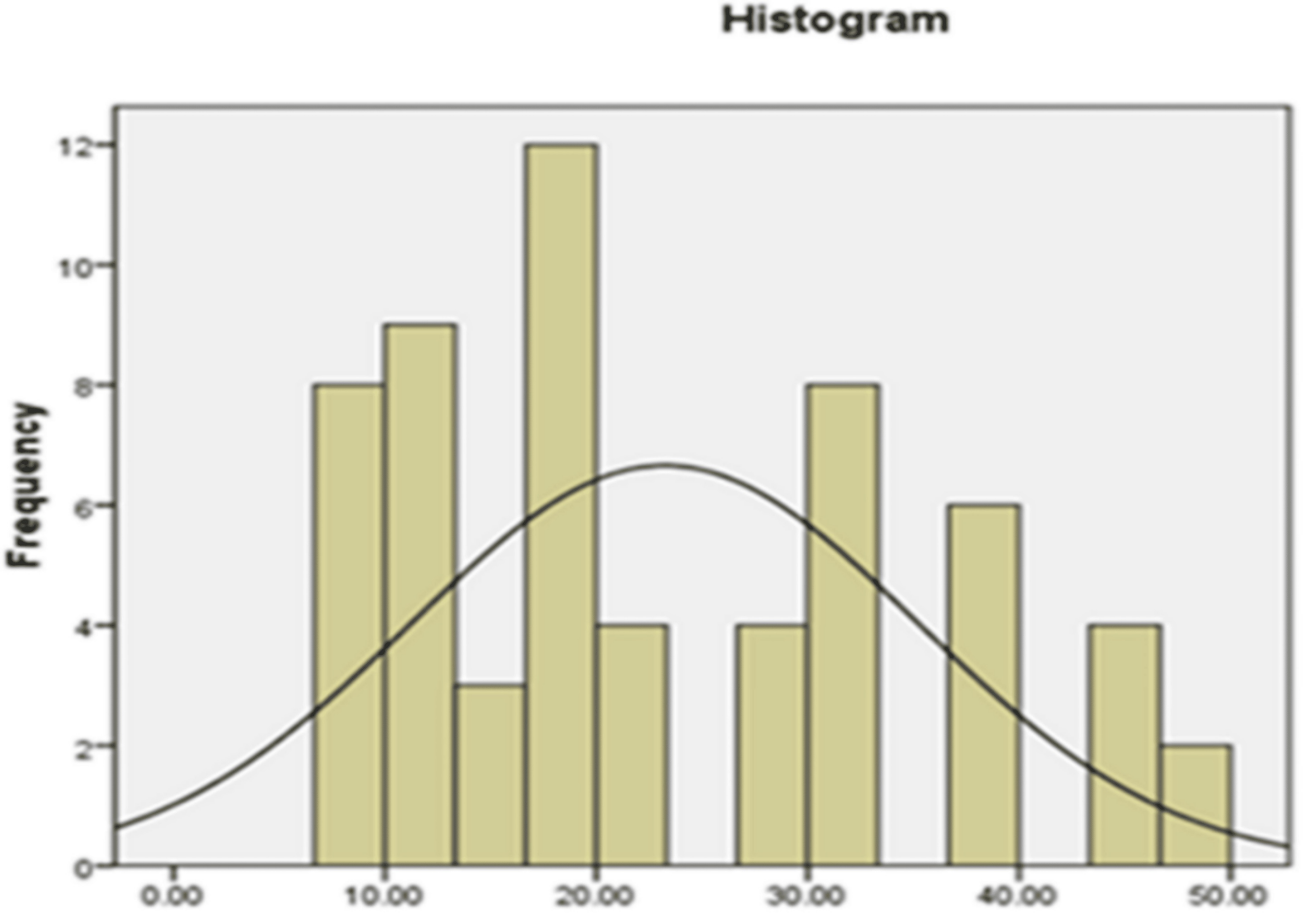
Histogram chart to check the normality of data distribution in the fear of negative evaluation variable

**Figure 3:**
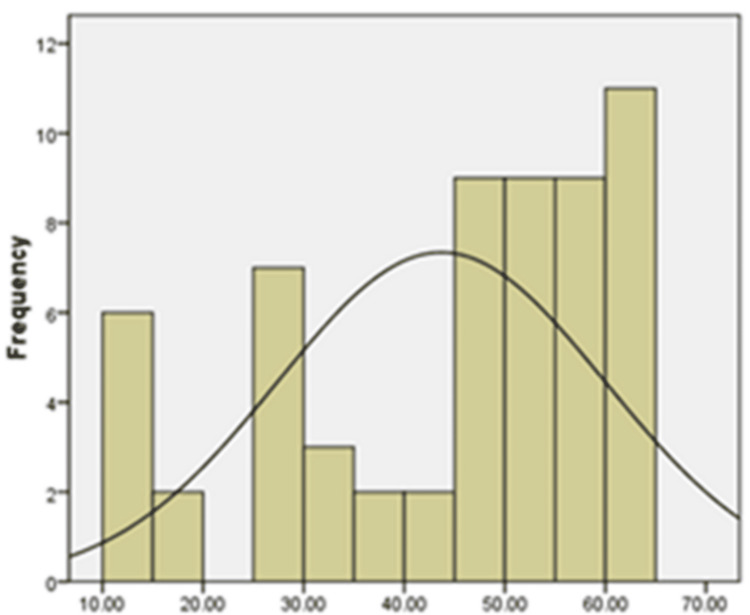
Histogram chart to check the normality of the data distribution in the shyness variable

Examining the bell-shaped distribution of the data in Figures 1-3 as well as the findings of the statistical studies pertaining to Table 3, which displays the outcome of dividing the skewness and kurtosis coefficients in mental health variables by the standard error Fear of negative assessment and shyness was between +2 and -2, it can be said that the data distribution is typical in mental health, FNE and shyness.

**Table 3:** Descriptive statistics of the variables under study in the research

Variables /statistics	Psychological health					Shyness	Fear of negative evaluation	Frequency (Percentage of participants)
	Depression	Anxiety	Stress	Total Psychological Health Score				
Mean	16.59	17.67	17.83		52.18	48.69	44.45	196 (100)
Standard Deviation	4.94	5.87	4.59		16.09	15.28	5.75	
Skewness	0.6	0.9`	0.5		0.21	0.29	0.47	
Kurtosis	0.43	0.35	0.20		0.98	0.83	0.63	

According to Table 4 results and considering that the test's significance level (p-value) was less than the 0.05 confidence level, it can be found that there is a significant relationship between university employees' mental health and its constituents (stress, anxiety, and depression) and shyness and fear of receiving a negative evaluation. Additionally, these concepts are able to predict mental health and its elements (stress, anxiety, and depression) in the studied society. In terms of correlation strength, the relationship between mental health and FNE and shyness is in a favorable situation.

**Table 4:** Results of calculating the Pearson correlation coefficient to predict the mental health of university staff P < 0.05

Criterion variable and its components	Predictor variable	Correlation coefficient	P-value
Depression	Shyness	0.638	0.01
	Fear of negative evaluation	0.459	0.04
Anxiety	Shyness	0.619	0.000
	Fear of negative evaluation	0.562	0.01
Stress	Shyness	0.555	0.01
	Fear of negative evaluation	0.560	0.03
Mental health	Shyness	0.697	0.000
	Fear of negative evaluation	0.597	0.01

The statistical analysis related to Table 5 shows that the significance level of the test is less than 0.05. Therefore, it can be said that mental health and its components (depression, anxiety and depression) have a significant relationship with the FNE and shyness in university employees. Considering the rounded coefficients for the multiple regression coefficients in Table 5, shyness and FNE as two predictive variables were able to explain 54% of the mental health criterion variable simultaneously. These two predictor variables were able to explain 41%, 43%, and 39% of mental health components (depression, anxiety, and stress) respectively. Therefore, according to the amount of explanation that the predictive variables shyness and FNE have from the criterion variable “mental health” and the components of “depression,” “anxiety,” and “stress” and considering the degree of correlation that exists Among them, it can be concluded that shyness and FNE can predict “mental health” and the components of “depression,” “anxiety,” and “depression” in university employees.

**Table 5:** Multiple regression analysis results for predicting mental health

Variable and its components	Predictor variable	Not standardized coefficients		F (contribution of the regression model to the residuals in the expression of total dispersion)	T (hypothesis test of coefficients)	Multiple regression coefficient	P-value	Variance explained
		Beta Coefficient (B)	Standard error					
Depression	Shyness	0.556	14.3458	70.574 (P-value: 0.03)	8.481	0.615	0 01	41%
	Fear of negative evaluation	0.166	9.06200		2.289		0.04	
Anxiety	Shyness	0.445	13.86567	79/857 (P-value: 0.000)	6.972	0.674	0.000	43%
	Fear of negative evaluation	0.316	12.14726		4.958		0.01	
Stress	Shyness	0.355	12.57328	64/601 (P-value: 0.02)	5.314	0.633	0,01	39%
	Fear of negative evaluation	0.364	12.61456		5.460		0.03	
Mental Health	Shyness	0.528	13.24745	118/177 (P-value: 0.000)	9.136	0.742	0,000	54%
	Fear of negative evaluation	0.306	11.42142		5.288		0.01	

## Discussion

Prediction of the mental health of university employees based on shyness and FNE was discussed in this study. The findings indicated that shyness and FNE can predict “mental health” and its sub-components (depression, anxiety, and stress) in university employees. This finding of the present research is in line with the results of separate studies conducted by concluded in their research that mental health has a significant relationship with body esteem, shyness, and FNE in students. The results of Liu [20] showed that negative self-evaluation and negative feedback have a negative and significant impact on social interactions and relationships and lead to social anxiety. It was found in Bober's [21] research that shy people have stress in communicating with others and numerous communication and avoidance challenges in most cases due to being shy and having different personality traits. Also, considering these consequences, it is highly likely that this group of people will suffer from rumination and negative self-evaluation. In another study, Margo [22] found that FNE can predict social anxiety and addictive behaviors. Also, Nonterah [23] reached this conclusion in another study that the FNE leads to an increase in depression, anxiety, and depression in the studied community. In addition, two types of research conducted in Iran are mentioned to support the results aligned with this research, these researches include separate research by Moghadam [24] and Maleki [25]. They showed in these studies that shyness, neuroticism, and effortful control are important factors in explaining and predicting social anxiety.

In the explanation of the findings of the present research, it should be said that people who have a negative evaluation of themselves and others, compared to people who are optimistic about their life, their abilities, the people around them, and their relationships, have problems in establishing and improving interpersonal relationships. And they suffer a lot of anxiety and stress. As a result of this result, this group of people faces a decrease in self-esteem, experience a decrease in performance, and as a result, suffer from depression. People's misunderstanding of how they are evaluated by others leads them to worry and anxiety [26,27]. Facing people's stressful events in the work environment and how to respond to them can be effective in their involvement in various anxiety disorders, depression, and social isolation. So that adopting destructive approaches in response to everyday issues in the living and working environment causes the emergence of disabilities in people's performance and reduces their self-esteem, which in turn leads to the rejection of this group of people by the members. Family, friends, and surrounding people and it leads to their withdrawal [28]. Shyness and withdrawal due to the fear it creates in people when engaging in social interactions, causes a decrease in social participation and an increase in self-criticism, and also results in the deterioration of the quality level of interactions. In addition, the extreme and obsessive increase in the aspect of caution in social interactions, which is one of the consequences of shyness, has no result other than abnormal cognitive, emotional, physiological, and behavioral changes that lead to fear and anxiety and the occurrence of avoidance behaviors.

Limitations

This study has several limitations that should be acknowledged. The cross-sectional design restricts the ability to establish causal relationships or assess long-term effects, as data are collected at a single point in time. Additionally, the reliance on self-report questionnaires may introduce response biases, affecting the accuracy of the findings. The sample, limited to university employees from one institution, may not be representative of broader populations, potentially limiting the generalizability of the results. Furthermore, the study does not account for all potential confounding variables, such as personal life events or other psychological conditions, which could influence mental health. Finally, the absence of qualitative data means that the nuanced experiences of participants may not be fully captured. Future research should consider longitudinal designs, incorporate qualitative methods, and include diverse samples to provide a more comprehensive understanding of the dynamics between self-esteem, FNE, and mental health.

## Conclusions

Based on the findings, it can be said that “mental health” and its components, i.e., “anxiety,” “stress,” and “depression” have a significant relationship with FNE and shyness in university employees. In this context, it can be said that FNE and shyness can predict “mental health” and its components in the studied society. Based on this, it is necessary to hold workshops to increase communication skills to achieve competencies. Career and social for academic staff have become more than before. Providing opportunities for academic staff to learn effective skills and strategies in facing job and communication challenges at work should be included in the in-service programs of universities. Self-awareness training programs for university employees should be prioritized and emphasis should be placed on accepting their strengths and weaknesses by themselves, which can minimize the possibility of fear, worry, stress, and anxiety caused by the evaluations of others and strengthen self-criticism capacities. And it leads to the development of interpersonal relationships in the work and family environment for employees. In the research fields, it is also suggested to conduct research with educational aspects for employees in order to increase self-knowledge and awareness of important aspects of personality. It is also recommended to conduct studies on the correlation between job satisfaction and mental health, FNE, and shyness among university employees.

## Figures and Tables

**Figure 4:**
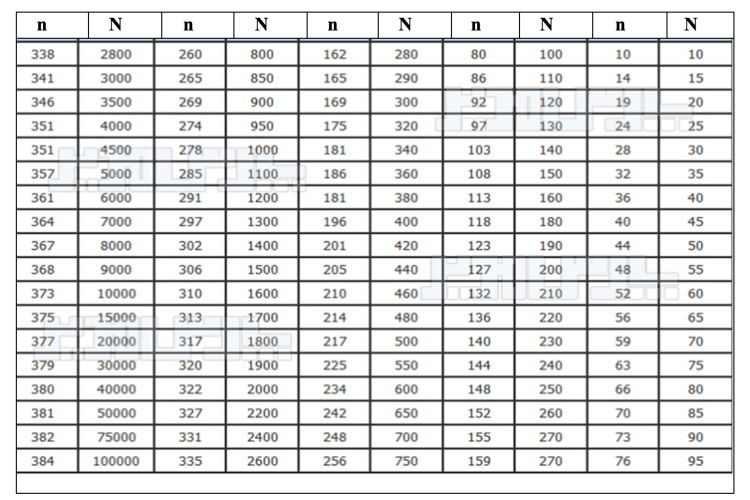
Kerjcie and Morgan determining sample size for research activities

## Appendices

**Table 6:** Depression Anxiety Stress Scale (DASS) by Lovibond and Lovibond (1995)

Row	Sentences	Options			
		Not at all	Low	Moderate	High
1	It is difficult for me to relax.				
2	I have noticed that my mouth is dry.				
3	I don't think I can experience any kind of good feeling.				
4	It is difficult for me to breathe.				
5	It is difficult for me to take the lead in doing work.				
6	I react excessively to my situations.				
7	I feel trembling in my body				
8	I feel that I consume a lot of mental energy.				
9	I am worried that I might get scared in some situations I clap stupidly.				
10	I feel like I have nothing to look forward to.				
11	I feel confused and confused.				
12	It is difficult for me to be calm and to live in peace.				
13	I feel dead and broken hearted.				
14	I am impatient and impatient with anything that prevents me from working.				
15	I feel that I may panic at any moment.				
16	I am not able to show enthusiasm about many things.				
17	I feel that I do not have much value as a person.				
18	I think I am very sensitive and easily hurt.				
19	Even if I do any physical activity, I have noticed that my heart is working abnormally (for example, a strong heartbeat or it stops working for a few moments).				
20	I feel fear for no valid reason.				
21	I feel that life is meaningless.				

**Table 7:** Scale of fear of negative evaluation by Watson and Friend (1969)

Row	Phrases	Options				
		I'm not like that at all	I am a little like this	I am almost like this	I am very much like this	I am very much like this
1	Even when I know I'm okay, I worry about what other people think of me					
2	Even when I know that others have an unfavorable opinion about me, I still don't worry.					
3	I am always afraid that others will notice my weaknesses.					
4	I rarely worry about the effect it has on others					
5	I am afraid that others will not agree with me					
6	I am afraid that others will find problems in me					
7	I don't care what other people think about me.					
8	When I'm talking to someone, I worry about what they think of me					
9	I usually worry about the effect I have on others					
10	Knowing that someone is judging me does not affect me much					
11	Sometimes I think I worry too much about what others think of me.					
12	I often worry that I might say the wrong thing or do the wrong thing.					

**Table 8:** Cheek and Bass’s Shyness Scale (1983)

Row	Sentences	Options				
		strongly disagree	Uncharacteristic	Neutral	Characteristic	strongly agree
1	I feel tense when I'm with people I don't know well.					
2	During the conversation with new acquaintances, I worry about saying funny and stupid things.					
3	I am socially motivated to some extent.					
4	It is difficult for me to ask other people to get information.					
5	I am often uncomfortable at parties and other social functions.					
6	When I'm in a group, it's hard for me to think about what I want to talk about.					
7	I feel comfortable even in unfamiliar social situations.					
8	It is difficult for me to act normal when I meet new people.					
9	I feel uncomfortable when I am around strangers.					
10	I have no doubts about my social competence.					
11	I get nervous when I talk to an influential person.					
12	It is difficult for me to look directly in the eyes of a person.					
13	I am usually the person who initiates the conversation.					
14	I usually worry if other people like to be with me or not.					
15	I sometimes feel physically uncomfortable when I am introduced to new people (for example, heart palpitations, sweating, itchy body, etc.).					
16	I don't understand that talking to strangers is difficult.					
17	I worry about how well I can fit in with new acquaintances.					
18	I am embarrassed to meet people of the opposite sex.					
19	It doesn't take long to overcome my shyness in new situations.					
20	I feel inhibited in social situations.					
